# Lipopolysaccharide-induced depression-like model in mice: meta-analysis and systematic evaluation

**DOI:** 10.3389/fimmu.2023.1181973

**Published:** 2023-06-08

**Authors:** Run Yin, Kailing Zhang, Yingming Li, Zilei Tang, Ruiyu Zheng, Yue Ma, Zonghan Chen, Na Lei, Lei Xiong, Peixin Guo, Gang Li, Yuhuan Xie

**Affiliations:** ^1^ College of Traditional Chinese Medicine, Yunnan University of Chinese Medicine, Kunming, China; ^2^ Basic Medical School, Yunnan University of Chinese Medicine, Kunming, China; ^3^ Academic Affairs Department, Yunnan University of Chinese Medicine, Kunming, China; ^4^ School of Clinical Medicine, Yunnan University of Chinese Medicine, Kunming, China; ^5^ Key Laboratory of Aromatic Chinese Herb Research, Yunnan Provincial University, Kunming, China; ^6^ Yunnan Innovation Team of Application Research on Traditional Chinese Medicine Theory of Disease Prevention, Yunnan University of Chinese Medicine, Kunming, China; ^7^ College of Ethnic Medicine, Yunnan University of Chinese Medicine, Kunming, China

**Keywords:** mouse model, lipopolysaccharide, systematic review, meta analysis, depression

## Abstract

Depression is a complex and biologically heterogeneous disorder. Recent studies have shown that central nervous system (CNS) inflammation plays a key role in the development of depression. Lipopolysaccharide (LPS)-induced depression-like model in mice is commonly used to studying the mechanisms of inflammation-associated depression and the therapeutic effects of drugs. Numerous LPS-induced depression-like models in mice exist and differ widely in animal characteristics and methodological parameters. Here, we systematically reviewed studies on PubMed from January 2017 to July 2022 and performed cardinal of 170 studies and meta-analyses of 61 studies to support finding suitable animal models for future experimental studies on inflammation-associated depression. Mouse strains, LPS administration, and behavioral outcomes of these models have been assessed. In the meta-analysis, forced swimming test (FST) was used to evaluate the effect size of different mouse strains and LPS doses. The results revealed large effect sizes in ICR and Swiss mice, but less heterogeneity in C57BL/6 mice. For LPS intraperitoneal dose, the difference did not affect behavioral outcomes in C57BL/6 mice. However, in ICR mice, the most significant effect on behavioral outcomes was observed after the injection of 0.5 mg/kg LPS. Our results suggests that mice strains and LPS administration play a key role in the evaluation of behavioral outcomes in such models.

## Highlights

- Neuroinflammation has been strongly implicated in the pathogenesis of depression.
- Lipopolysaccharide (LPS) administration induced depression-like model in mice is commonly used to studying the mechanisms of inflammation-associated depression and the therapeutic effects of drugs.
- We systematically reviewed studies and performed cardinal of 170 studies and meta-analyses of 64 studies to support finding suitable animal models for future experimental studies on inflammation-associated depression.
- Mouse strains, LPS administration, and behavioral outcomes of these models have been assessed in meta-analysis.

## 1 Introduction

Depression is a global disease with a high incidence and suicide rate. The World Health Organization indicates that ~300 million people worldwide suffer from varying degrees of depression, affecting about 20% of the population and thereby creating a heavy social burden (1). The Global Burden of Disease, Injury, and Risk Factors Study showed that depression caused 34.1 million years of living with disability (YLD), ranking fifth among the causes of YLD (2017) (2). Mental health problems are more prominent in COVID-19 pandemic. Depressive symptoms and clinically significant depression are commonly reported among individuals as part of the post-COVID-19 syndrome (3). Therefore, research advances and application of new discoveries or techniques are imminent. The understanding of the pathophysiological mechanisms of depression has evolved over the last decades. Due to the complexity and heterogeneity of depression, it is challenging to determine its exact biological mechanisms, which are largely determined by genetic and environmental factors. So far, many hypotheses have been proposed for the pathogenesis of depression, including heritability, neurotransmitter systems, brain-derived neurotrophic factor (BDNF), and overactivity of the hypothalamic pituitary-adrenal axis (4), etc. Whereas existing antidepressants are mainly based on the monoamine hypothesis, a proportion of depressed patients (20% to 30%) do not respond to pharmacological treatment.

Since 1991, Smith (5) first proposed the neuroinflammation hypothesis of depression, increasingly more studies provide strong evidence to suggest that neuroinflammation is critical in clinical depression. Recent reviews have investigated that patients with depression are often accompanied by elevated levels of inflammatory markers, and persistent inflammatory activation can make susceptible people suffer from depression (6, 7). Grigoleit has reported that injection of the bacterial LPS in humans can cause an increase in plasmal pro-inflammatory cytokines and dose-related decreased mood (8), which suggests that inflammation is a causal factor in the subgroup of depression and not just an epiphenomenon (9). Meanwhile, the finding that the severity of depressive symptoms after COVID-19 syndrome has been shown to be proportional to the systemic inflammation measured at baseline during acute infection provides support for this hypothesis (10). Pharmacological studies have also confirmed the interconnection between depression and inflammation. Several antidepressants have been shown to reduce inflammation in both preclinical (11) and clinical (12, 13) models of neuropsychiatric disorders. Antidepressants such as selective serotonin reuptake inhibitors fluoxetine (14) and paroxetine, tricyclic antidepressants amitriptyline and clomipramine, and monoamine oxidase inhibitors tranylcypromine can prevent LPS-induced microglial changes and the production of inflammatory markers interleukin-1β (IL-1β), interleukin-6 (IL-6), and tumor necrosis factor-α (TNF-α) (15). These studies suggest that antidepressants may modulate neuroinflammation by modulating the microglial phenotype, which may be one of the mechanisms by which antidepressants work.

Based on the etiology of depression, a variety of unavoidable or uncontrollable stress exposure have been explored on animals to exhibit phenotypes that are similar to the symptoms of depressed patients (16). During the last 50 years, depression-like animal models have improved our understanding of the disease and have played a key role in the development of antidepressants (17). However, these models still have limitations in understanding the inflammatory mechanisms of depression and related antidepressant drug studies. *In vivo*, LPS acts as an inflammatory inducer that activates monocytes, macrophages, endothelial cells, and epithelial cells, which in turn activates cellular signaling systems leading to increased levels of various cytokines and inflammatory mediators(18). These peripheral inflammatory signals reach the central nervous system (CNS) through endothelial cells or second messengers *via* the blood–brain barrier, triggering neuroinflammation (19). Hence, peripheral or central injection of LPS can activate microglia and trigger a series of inflammatory responses to induce depression (15). In addition, the disruption of a subset of neurons that express parvalbumin interneuron mediates systemic inflammation–induced depression-like behavior and working memory impairment by LPS challenge (20). Animal models of inflammation-related depression are most commonly induced by administration of LPS. In the past 5 years, the number of these animal models has increased considerably. Among these mouse models, variations in factors such as animal strain, administration of LPS determine the face, construct and predictive validity offered in each model. However, this differs widely between models. It is critical to assess the validity of these models in terms of whether the animal strains and methodologies studied in these models are constructively valid for depression-like behavior. Therefore, we reviewed the experimental literature of the past five years to provide a comprehensive report of the different protocols and parameters used in these models.

## 2 Materials and methods

### 2.1 Search methodology and inclusion criteria

The present meta-analysis was performed according to the Preferred Reporting Items for Systematic Reviews and Meta-Analyses (PRISMA) guidelines (21). Two authors (Yingming Li and Zilei Tang) searched the PubMed database for articles published between January 1, 2017, and July 31, 2022, the following search terms were used “Depression [Mesh] OR depressive OR depressant AND lipopolysaccharide* AND mice* OR “ mouse”[Mesh]. In order to count the experimental parameters of LPS-induced depression in mice, the authors developed the following screening conditions:

(1) Articles are published as original articles.
(2) Experimental protocol of the LPS-induced depression model in mice is described.
(3) Mice without other co-morbidities, such as depression + obesity, depression + diabetes.
(4) Mice did not experience other stresses or injuries, such as early maternal separation, cerebral hemorrhage, etc.
(5) Mice used were not genetically edited.
(6) No LPS combined with other methods was used to induce depression model in mice, such as LPS + chronic unpredictable mild stress.

For the articles that met the above six criteria, the authors extracted the mouse strain, LPS dose, routes and times of administration, and behavioral test data for Chi-square test.

Based on serotype the consensus and the previous step of screening criteria, the authors developed the following inclusion criteria for meta-analysis:

(1) Forced swimming test (FST) was used to assess depressive behavior in mice.
(2) Experimental protocol using intraperitoneal injection of LPS.
(3) Experimental protocols used 0.5mg/kg or 0.83mg/kg or 1mg/kg as the induction dose of LPS.

### 2.2 Extraction of study data

For studies that met the inclusion criteria, the two authors (Run Yin and Yue Ma) extracted the following data in a table to facilitate subsequent frequency statistics and meta-analysis: (1) name of the first author and year of publication; (2) strain of mice; (3) dose and times of LPS administration; (4) behavioral test method selected; and (5) mean, standard deviation, and sample size (n) of the duration of immobilization in FST for mice in each model and control group. Data were extracted directly from the graphs or figures by using a digital scale. Any disagreements in data extraction were resolved by discussion with a third reviewer (kailing Zhang) until a consensus was reached. The meta-analysis was performed after collecting at least three studies from each group and, when available, providing the mean immobility time, the standard deviation, and sample size (22).

### 2.3 Chi-square test

The data obtained from the counting process is known as the count data. To test either the counts are differed significantly or not, the Chi-square test is applied (23). Statistical analyses were performed using IBM SPSS Statistics. Data (e.g., mouse strain, routes of LPS administration, times and dose of LPS administration, and LPS serotype) were summarized using frequencies and percentages and arranged in descending order of the number of times they were used. To identify significant of the experimental parameters chosen by the investigators between variables, Chi-squared analysis was performed (Separately tested for the presence of selection differences between two adjacent parameters). When an expected count less than 5, the Fisher’s Exact Test was used instead of the Chi-squared analysis. At a 95% confidence interval, values of *P* < 0.05 were deemed as statistically significant.

### 2.4 Assessment of risk of bias in included studies and publication bias

The risk of bias was assessed for each of the included studies by two authors independently using SYRCLE’s risk of bias tool. The answer to the assessment questions was either ‘‘yes” (indicating low risk of bias) or ‘‘no” (indicating high risk of bias). For unclear items, an ‘‘unclear” tag was assigned: random sequence generation (selection bias), baseline characteristics (selection bias), allocation concealment (selection bias), random housing (performance bias), blinding of participants (performance bias), random outcome assessment (detection bias), blinding of outcome assessment (detection bias), incomplete outcome data (attrition bias), selective reporting (reporting bias) and other bias. The publication bias was assessed *via* Begg’s test by Stata software (Version 12.0). Duval and Tweedie’trim and fill’ was used to adjust the analysis for the effects of publication bias.

### 2.5 Data synthesis

To assess the effect of LPS inducing depression-like behavior in mice, we pooled the immobility time of FST in mice from all the included studies. Forest plots generated from the data were graphically analyzed and visualized using RevMan 5.3 software. A random-effect model was used for the analysis and the standard mean difference (SMD) was considered. To evaluate the effect of treatment on each parameter, 95% confidence interval (CI) was used and significance set at *P* < 0.10. SMD is a measure of the effect size, and the effect size reflects the degree of difference between the LPS group and the control group. Heterogeneity values were also calculated to determinate if included studies were suitable for meta-analysis. I^2^ has been used to quantify heterogeneity and I^2^ > 50 was considered substantial and significant if *P* < 0.10. Sensitivity analysis was also performed to assess heterogeneity of the study results by excluding each study in turn, for each of the parameters considered.

## 3 Results

### 3.1 Literature search and study characteristics

**Figure 1:**
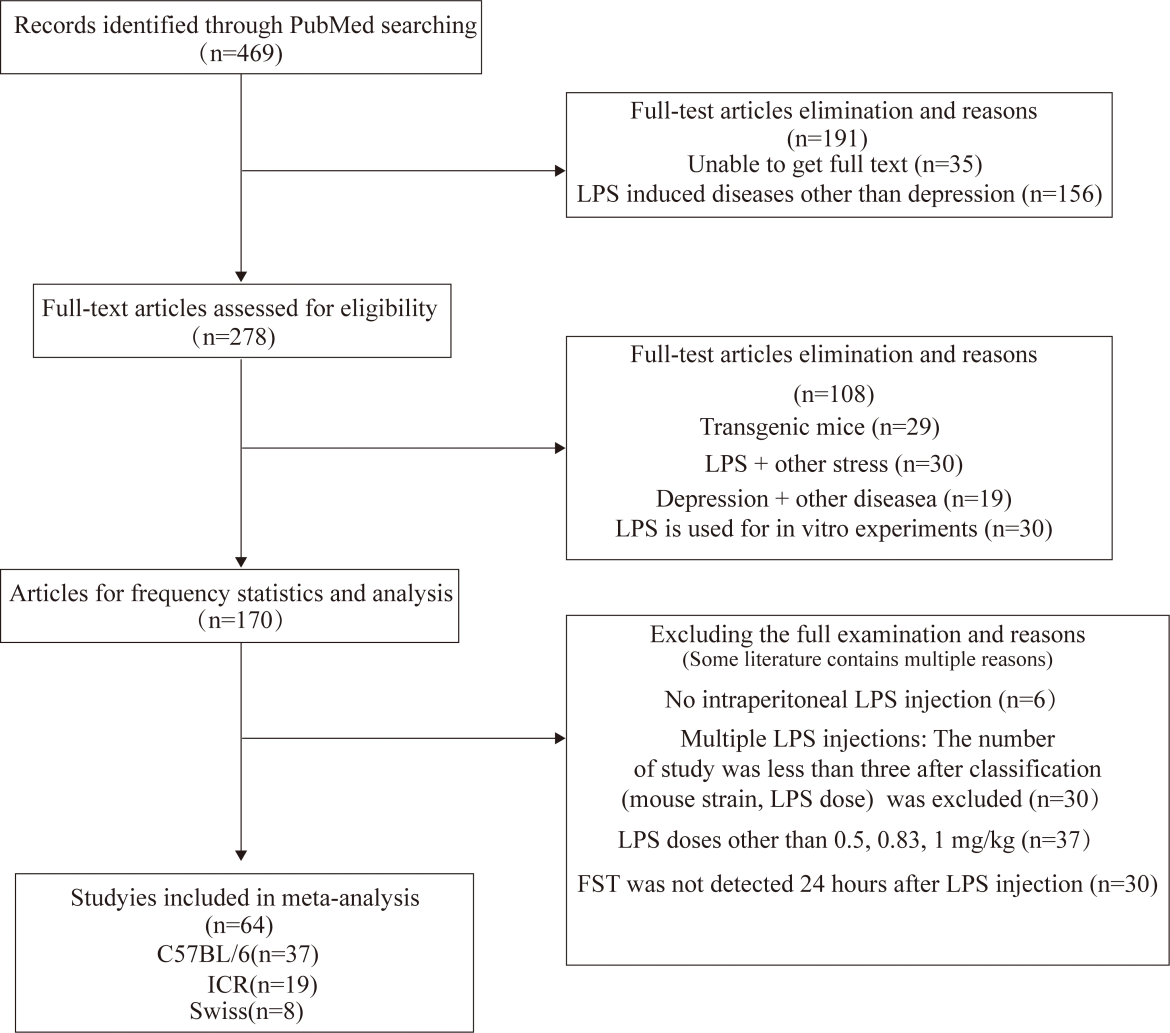
Flowchart of screening and choosing eligible articles.

A total of 469 studies published from 2017 to 2022 were retrieved from the PubMed database and 170 studies were left after excluding those that did not meet the inclusion criteria. The literature was based on mouse strain, behavioral tests, LPS (dosage, routes and times of administration) statistics, and meta-analysis of FST in depressive-like mice. The flowchart of screening and choosing eligible articles are shown in **Figure 1**.

### 3.2 Counts results for mice strains, LPS administration and behavior test

#### 3.2.1 Mice strains

Mouse strains such as C57BL/6, ICR, Swiss, and Balb/c have been widely used in preclinical studies of depression. Among the 170 studies, there are 2 studies did not specify the mouse strain. Statistical results (**Table 1**) showed that C57BL/6 mice were the most frequently used strain in the LPS-induced depression model (frequency 86, accounting for 51.19%; vs. ICR, *P* = 0.000), followed by ICR mice (frequency 40, accounting for 23.81%; vs. Swiss, *P* = 0.011) and Swiss mice (frequency 22, accounting for 13.10%; vs. Balb/c, *P* = 0.070), Balb/c mice, CD-1, Kunming, and Wistar albino mice were the last in order; we did not perform statistical analyses because few studies have used these strains. Therefore, we subsequently selected studies that used C57BL/6 mice, ICR mice, and Swiss mice for further meta-analysis.

**Table 1:** Mice strains.

Mice strain	NO. of studies using mice	Use this strain			*p*-value
		YES	NO	Percentage(%)	
C57BL/6	168	86	82	51.19	0.000
ICR	168	40	128	23.81	0.011
Swiss	168	22	146	13.10	0.070
Balb/c	168	12	156	7.14	0.017
NMRI	168	3	165	1.79	1.000
CD-1	168	2	166	1.19	1.000
Kunming	168	2	166	1.19	1.000
Wistar albino	168	1	167	0.6	

#### 3.2.2 Routes of LPS administration

The onset of depression may be induced by different molecular pathways due to the route of administration. The statistical results showed the following three ways of LPS administration: intraperitoneal injection (IP), intracerebroventricular injection (ICV), and intragastric (IG), among which IP was the most commonly used administration method (frequently 164, accounting for 96.47%, vs. ICV, *P* = 0.000) (as shown in **Table 2**).

**Table 2:** Routes of lipopolysaccharide administration.

Route of administration	NO. of studies	Use this route			*p*-value
		YES	NO	Percentage (%)	
IP	170	164	6	96.47	0.000
ICV	170	5	165	2.94	0.215
IG	170	1	169	0.59	

#### 3.2.3 Times of LPS administration

The statistical results showed that the administration times of LPS-induced depressive-like behavior in mice included single administration and multiple injections of LPS to induce depression. A total of 2 administration regimens were used in 3 studies. Among them, single induction of depression in mice was the most common experimental method (frequency 139, accounting for 80.35%, vs. repeatedly, *P* = 0.000) (As shown in **Table 3**).

**Table 3:** Times of lipopolysaccharide administration.

Times of administration	NO. of protocols	Use this protocol			*p*-value
		YES	NO	Percentage (%)	
Once	173	139	34	80.35	0.000
Repeatedly	173	34	139	19.65	

#### 3.2.4 LPS dose and serotype

Different doses of LPS may have an effect on the degree of depression in mice. Based on the statistical results of the LPS administration route and times, we divided the dose of LPS into an IP/single injection, IP/multiple injections/single dose, IP/multiple injections/multi-dose, and ICV/single injection to determine the frequency statistics.

The results of IP/single injection showed that 0.83 mg/kg was the most frequently used dose (frequency 60, accounting for 43.48%, vs. 0.5 mg/kg, *P* = 0.000), followed by 0.5 mg/kg (frequency 23, accounting for 16.67%) and 1 mg/kg (frequency 18, accounting for 13.04%), and no significant difference was found in the dose selection between the two (*P* = 0.397), details of other doses are provided in **Table 4**.

**Table 4:** Dose of lipopolysaccharide for a single intraperitoneal injection.

LPS Dose (mg/kg)	NO. of studies using dose	Use this dose			*p*-value
		YES	NO	Percentage (%)	
0.83	138	60	78	43.48	0.000
0.5	138	23	115	16.67	0.397
1	138	18	120	13.04	0.068
0.8	138	9	129	6.52	0.273
2	138	5	133	3.62	1.000
0.1	138	4	134	2.90	1.000
0.3	138	4	134	2.90	1.000
5	138	4	134	2.90	1.000
1.2(including 1.25)	138	3	135	2.17	1.000
0.4	138	2	136	1.45	1.000
0.083	138	1	137	0.72	1.000
0.25	138	1	137	0.72	1.000
0.6	138	1	137	0.72	1.000
0.85	138	1	137	0.72	1.000
1.5	138	1	137	0.72	1.000
1.8	138	1	137	0.72	

The results of the IP/multiple injections/single dose (**Table 5**) was similar to those of a single administration. The three most frequently used doses were 0.5 mg/kg (frequency 8, accounting for 29.63%, vs. 0.83 mg/kg, *P* = 0.535), 0.83 mg/kg (frequency 6, accounting for 22.22%, vs. 1 mg/kg, *P* = 1.000), and 1 mg/kg (frequency 6, accounting for 22.22%). No significant difference was found among these three doses. For the experimental design of multiple injections in a single dose, the most used protocol was “1 mg/kg, once a day for 5 days.”

**Table 5:** Single dose of multiple intraperitoneal injections of lipopolysaccharide.

LPS Dose (mg/kg)	NO. of studies using dose	Use this dose			*p*-value	Study Design
		YES	NO	Percentage (%)		
0.5	27	8	19	29.63	0.535	Once a day for 10 days
						Once a day for 6 days
						Once a day for 7 days
						Once a day for 4 days
0.83	27	6	21	22.22	1.000	Once a day for 5 days
						Once a day for 3 days
						Once a day for 2 days
						The interval between injections was 16 h
1	27	6	21	22.22	0.250	Once a day for 5 days
						Once a day for 2 days
0.1	27	2	25	7.41	1.000	Once a day for 4 days
						Twice a week for 21 days
2	27	2	25	7.41	1.000	Once a day for 3 days
1.2	27	1	26	3.70	1.000	Once a day for 7 days
0.083	27	1	26	3.70	1.000	Once a day for 4 days
0.25	27	1	26	3.70		Once a day for 7 days

A total of four methods were used for LPS multi-dose modeling (**Table 6**), and the most commonly used methods were “0.052, 0.104, 0.208, and 0.415, 0.83 mg/kg; one dose per day for 5 days.” Finally, we obtained 5 studies that used the intracerebral injection of LPS. The location and volume of injections are described in detail in **Table 7**.

**Table 6:** Multiple doses of lipopolysaccharide for intraperitoneal injection.

Study design
(1) Day 1: 0.75 mg/kg; day 2: 1 mg/kg; day 3: 1.25 mg/kg; day 4: 1 mg/kg; day 5: 0.75mg/kg (2) 0.052, 0.104, 0.208, 0.415, 0.83 mg/kg; One dose per day for 5 days; (3) Day 1: 0.2 mg/kg; day 6: 8.3 mg/kg; (4) Week 1-2: 0.33 mg/kg; week 3: 0.53 mg/kg; week 4: 0.63 mg/kg; week 5: 0.73 mg/kg; week 6: 0.83 mg/kg;

**Table 7:** Dose of lipopolysaccharide for a single intracerebral injection.

Concentration	Injection volume/quality	Injection location
10 mg/mL	1 µL	ML: -1.0 mm; AP: -0.5 mm; DV: -2.5 mm;
10 ng/µL	1 µL/side	/
/	10 µg	AP: -0.22 mm; ML: + 1.0 mm
/	100 ng	ML: +1.0 mm; AP: 0.5 mm; DV: 2.0 mm
10 mg/mL	1 µL	ML: -1.0 mm; DV: -2.5 mm; AP: -0.5 mm

The doses to be used should depend on the LPS serotype as different serotypes have different endotoxin activity. A total of 6 serotypes of LPS were used in the included studies (**Table 8**), and 68 studies did not specify the LPS serotype. Among them, 055: B5 was the most frequently used LPS serotype (frequency 35, accounting for 34.31%, vs. 0127: B8, P = 0.766), followed by 0127: B8 (frequency 33, accounting for 32.35%, vs. 0111: B4, *P* = 0.444) and 0111: B4(frequency 28, accounting for 27.45%), which indicated that there was no statistically significant difference in the selection of the LPS model among these three types. The most frequently used dosage of 0127: B8, 0111: B4, 026: B6 was 0.83mg/kg, while 055: B5 was 0.5mg/kg.

**Table 8:** Lipopoysaccharide serotypes.

LPS serotypes	NO. of studies using serotypes	Use this serotypes			*p*-value
		YES	NO	Percentage (%)	
055: B5	102	35	67	34.31	0.766
0127: B8	102	33	69	32.35	0.444
0111: B4	102	28	74	27.45	0.000
026: B6	102	3	99	2.94	1.000
RH51487	102	2	100	1.96	1.000
L-7985	102	1	101	0.98	

#### 3.2.5 Behavior test

Behavioral test is acknowledged and intuitive method to test whether the animal model of depression was successfully established. A total of 18 behavioral methods were used in our results, among which the most commonly used methods were FST (frequency 140), suspension tail test (TST) (frequency 115), open field test (OFT) (frequency 95), and sucrose preference test (SPT) (frequency 78), the remaining items are presented in **Table 9**.

**Table 9:** Behavioral test.

Behavioral tests	Frequency	Behavioral tests	Frequency
FST	140	SIT	3
TST	115	LDB	3
OFT	95	YMT	3
SPT	78	MWM	2
LMA	19	Rotarod	2
EPM	12	PAT	2
NSFT	11	FUST	2
ST	8	ATM	2
NORT	4	TFC	1

FST, Forced Swimming Test; TST, Tail Suspension Test; OFT, Open Field Test; SPT, Sucrose Preference Test; LMA, The locomotor activity; EPM, Elevated plus maze test; NSFT, Novelty suppressed-feeding test; ST, Splash Test; NORT, Novel object recognition task; SIT, Social interaction test; LDB, Light-dark box; YMT, Y maze test; MWM, Morris water maze test; PAT, Passive avoidance test; FUST, Female urine sniffing test; ATM, Autonomic activity tests; TFC, Trace fear conditioning.

### 3.3 Meta-analysis

However, the highest frequency of use is not considered to be the optimal choice of experimental parameters. A meta-analysis was performed to assess the effects of different LPS doses on depressive behavior in different mouse strains using FST. Based on the frequency results shown in 3.2, we used C57BL/6 mice, ICR mice, and Swiss mice as observational subjects to determine the immovability time of FST after IP injection of LPS (0.5 mg/kg, 0.83 mg/kg, and 1 mg/kg). The results indicated that the mice showed a state of behavioral despair after LPS injection. This was evidenced by an increased FST immobility time in the LPS group compared with the control group.

#### 3.3.1 Risk of bias in included studies and publication bias

**Figure 2:**
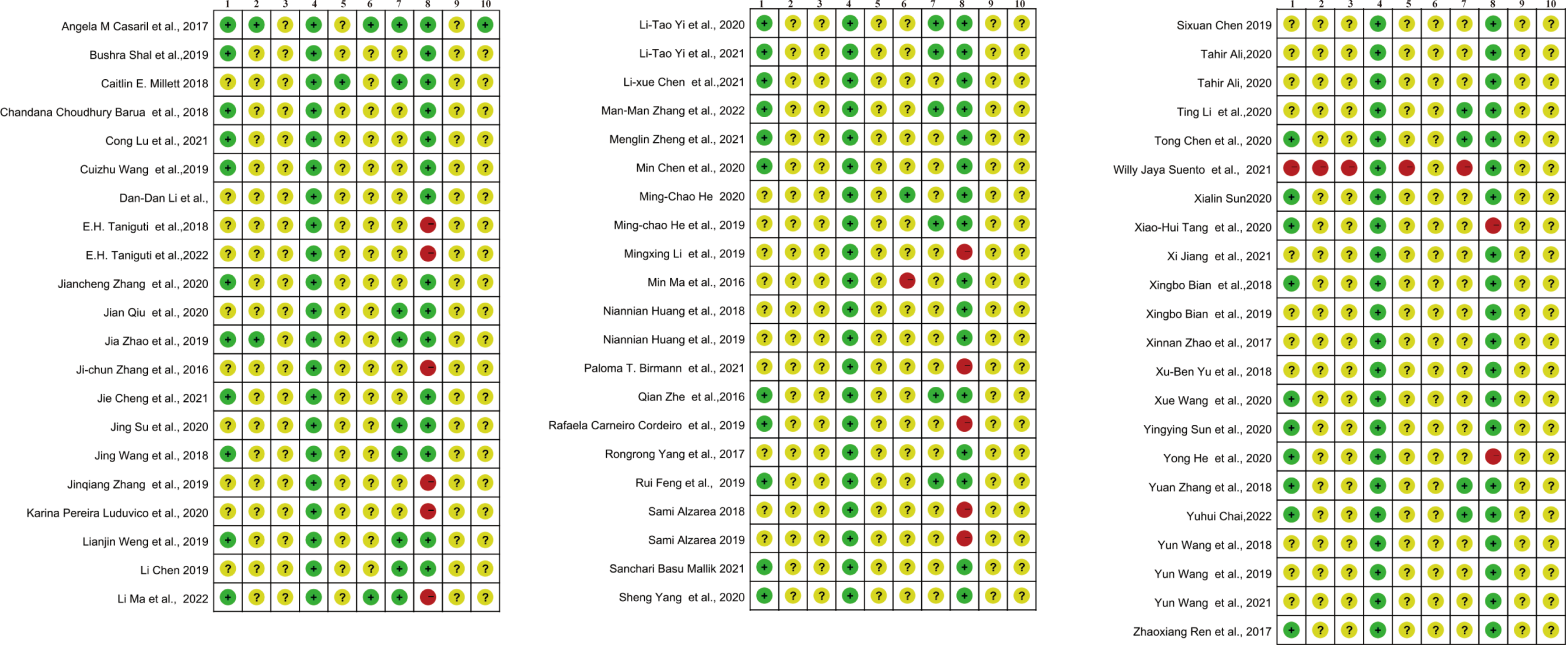
Risk of bias summary for the included studies. 1, Random sequence generation (selection bias). 2, Baseline characteristics (selection bias). 3, Allocation concealment (selection bias). 4, Random housing (performance bias). 5, Blinding of participants (performance bias). 6, Random outcome assessment (detection bias). 7, Blinding of outcome assessment (detection bias). 8, Incomplete outcome data (attrition bias). 9, Selective reporting (reporting bias). 10, Other bias.

The risk of bias for each included study is summarized in **Figure 2**. Of the 64 included studies, 32 described the methods used to generate the allocation sequence and one study (24) explicitly described no randomization was performed, while the remaining studies lacked information about this process. Only 1 study reported baseline characteristics other than age, sex, and weight, such as locomotion pattern, unusual respiratory, piloerection, etc. (25). No study clarified whether the allocation of different groups was sufficiently hidden. The breeding conditions and environment of all experimental animals included in the study were the same; therefore, we considered that the animal placement complied with the principle of randomization (26). Only one study reported that animal breeders and/or researchers were blinded to the study groups, although it did not report the specific processes (27). Three and 19 studies reported randomization and blinding of outcome evaluation respectively. In terms of incomplete outcome data, in 13 studies, animals were withdrawn during the experimental procedure, although the impact of withdrawals on the results was not examined. Selective outcome reporting bias was assessed as unclear for all studies because none reported using a research protocol defining primary and secondary outcomes. No study was identified with other problems that could result in high risk of bias.

**Figure 3:**
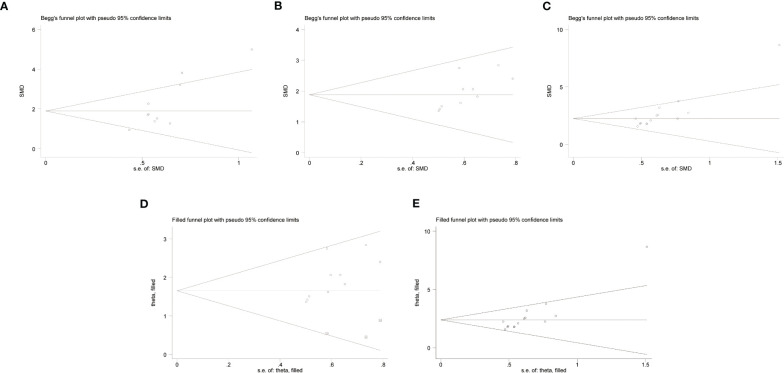
Begg’s bias test of **(A)** C57BL/6 -0.83 mg/kg LPS. **(B)** C57BL/6 - 1 mg/kg LPS. **(C)** ICR - 0.83 mg/kg LPS. Trim-and- fill evaluation of **(D)** C57BL/6 - 1 mg/kg LPS. **(E)** ICR - 0.83 mg/kg LPS.

The risk of publication bias is shown in a funnel plot graph (**Figure 3**). The Begg’s bias test showed that, except for”C57BL/6-0.83 mg/kg LPS” (Pr > |z| = 0.074 > 0.05) (**Figure 3A**), publication bias was detected for “C57BL/6 - 1 mg/kg LPS” (Pr > |z| = 0.032 < 0.05) (**Figure 3B**) and “ICR - 0.83 mg/kg LPS” (Pr > |z| = 0.001 < 0.05) (**Figure 3C**). However, when these publication biases were corrected using trim and fill method by adding theoretically missing studies, the results did not change significantly [(**Figures 3D, E**). We did not conduct a publication bias test for the other outcome measures because of the small number of studies (<10)].

#### 3.3.2 Mice strains

**Figure 4:**
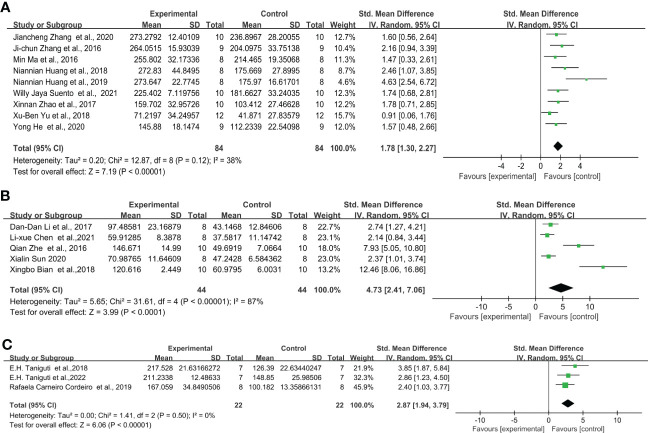
Forest plots of standardized mean differences in forced swimming test (s) in **(A)** C57BL/6 mouse - 0.5 mg/kg LPS. **(B)** ICR mouse-0.5 mg/kg LPS. **(C)** Swiss mouse - 0.5 mg/kg LPS.

Under the protocol of 0.5 mg/kg LPS single injection, the study includes nine articles from the C57BL/6 strain, five articles from the ICR strain, and three articles from the Swiss strain. Overall, 0.5 mg/kg LPS injection significantly increased the immobility time in the FST of C57BL/6, ICR, and Swiss mice. In addition, C57BL/6 mice (**Figure 4A**) (SMD = 1.78 [1.30, 2.27], Z =7.19 (*P* < 0.00001) and heterogeneity χ^2^ = 12.87, *P* = 0.12, I^2^ = 38%) exhibit smaller overall effect size compared to ICR (**Figure 4B**) (SMD = 4.73 [2.41, 7.06], Z =3.99 (*P* < 0.00001) and heterogeneity χ^2^ = 31.61, *P* < 0.00001, I^2^ = 87%) and Swiss mice (**Figure 4C**) (SMD = 2.87 [1.94, 3.79], Z =6.06 (*P* < 0.00001) and heterogeneity χ^2^ = 1.41, *P* = 0.5, I^2^ = 0%). Sensitivity analysis showed that none of the study reversed the effect identified by the meta-analysis, and excluding individual studies in order did not descend heterogeneity in ICR strain.

**Figure 5:**
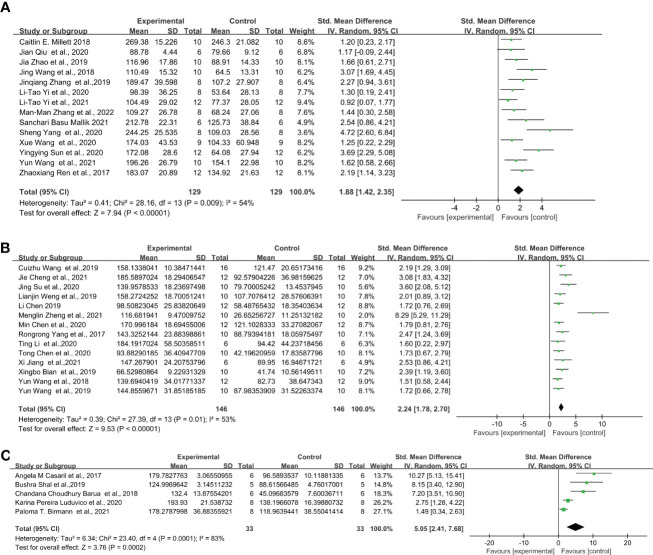
Forest plots of standardized mean differences in forced swimming test (s) in **(A)** C57BL/6 mouse - 0.83 mg/kg LPS. **(B)** ICR mouse- 0.83 mg/kg LPS. **(C)** Swiss mouse-0.83 mg/kg LPS.

Similarly, for the protocol of 0.83 mg/kg LPS single injection, C57BL/6 strain showed even lower effect sizes. The overall effect size for LPS-induced mice after FST was the following: C57BL/6 mice (**Figure 5A**) (SMD = 1.88 [1.42, 2.35], Z =7.94 (*P* < 0.00001) and heterogeneity χ^2^ = 28.16, *P* = 0.009, I^2^ = 54%), ICR mice (**Figure 5B**) (SMD = 2.24 [1.78, 2.70], Z =9.53 (*P* < 0.00001) and heterogeneity χ^2^ = 27.39, *P* = 0.01, I^2^ = 53%), Swiss mice (**Figure 5C**) (SMD = 5.05 [2.41, 7.68], Z =3.76 (*P* = 0.0002) and heterogeneity χ^2^ = 23.4, *P* = 0.00001, I^2^ = 83%). Among them, C57BL/6, ICR and Swiss mice showed significant heterogeneity. The sensitivity analysis proved that the heterogeneity originated from a single study (28–30) of C57BL/6 and ICR, respectively. and after excluding this study heterogeneity χ^2^ = 20.46, *P* = 0.06, I^2^ = 41%, χ^2^ = 10.95, *P* = 0.53, I^2^ = 0%, and did not have a significant effect on the overall effect value (SMD = 1.65 [1.26, 2.05], Z =8.20 (*P* < 0.00001); SMD = 2.06 [1.75, 2.37], Z =13.03 (*P* < 0.00001)) in C57BL/6 and ICR mice. However, no source of heterogeneity was found in Swiss mice.

#### 3.3.3 LPS doses

**Figure 6:**
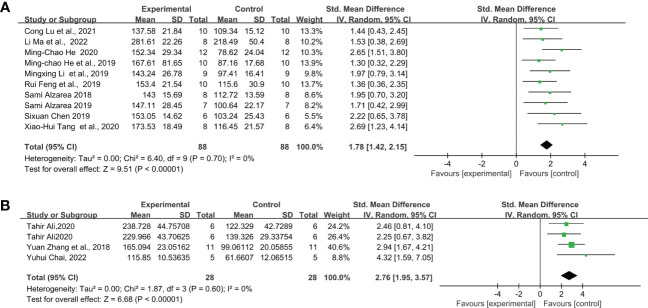
Forest plots of standardized mean differences in forced swimming test (s) in **(A)** C57BL/6 mouse - 1 mg/kg LPS. **(B)** C57BL/6-1mg/kg-Once a day for 5 days.

Through further analysis, different LPS doses did not exhibit induction differences in the FST of C57BL/6 mice, but in ICR and Swiss mice. When injected with 0.5 mg/kg (**Figure 4A**) (SMD = 1.78 [1.30, 2.27], Z =7.19 (*P* < 0.00001)), 0.83mg/kg (**Figure 5A**) (SMD = 1.88 [1.42, 2.35], Z =7.94 (*P* < 0.00001)) and 1mg/kg (**Figure 6A**) (SMD = 1.78 [1.42, 2.15], Z =9.51 (P < 0.00001) and heterogeneity χ^2^ = 6.40, P = 0.70, I^2^ = 0%) doses of LPS, the meta-analysis showed that the sizes of the overall effect of FST immobility time were at the same level in C57BL/6 mice. Whereas 0.5 mg/kg LPS-induced performance a greater effect size than 0.83 mg/kg in ICR (**Figures 4B**, **5B**) (SMD = 4.73 [2.41, 7.06], Z =3.99 (*P* < 0.00001), SMD = 2.24 [1.78, 2.70], Z =9.53 (*P* < 0.00001)). However, Swiss mice showed opposite results, 0.83 mg/k (**Figure 5C**) (SMD = 5.05 [2.41, 7.68], Z =3.76 (*P* = 0.0002)) LPS-induced performance a greater effect size than 0.5 mg/kg (**Figure 4C**) (SMD = 2.87 [1.94, 3.79], Z =6.06 (*P* < 0.00001)). Meta-analysis was not performed on ICR mice, Swiss mice induced by 1 mg/kg due to the limited number of articles.

Furthermore, we calculate the overall effect size of the LPS total dose applied during the intervention period. However, meta-analysis was only achieved under “C57BL/6-1mg/kg-Once a day for 5 days” due to the limited number of articles meeting the inclusion criteria. The result showed that five consecutive injections of 1mg/kg LPS (**Figure 6B**) (SMD = 2.76 [1.67, 4.21], Z =6.68 (*P* < 0.00001) and heterogeneity χ^2^ = 1.87, *P* = 0.60, I^2^ = 0% produced a greater effect size than a single injection of LPS in C57BL/6 mice (**Figure 6A**) (SMD = 1.78 [1.42, 2.15], Z =9.51 (*P* < 0.00001) and heterogeneity χ^2^ = 6.40, *P* = 0.70, I^2^ = 0%).

### 3.4 Summary

For mouse strains, C57BL/6 mice seem to be the optimal choice because they have lower heterogeneity and can more stably reflect behavioral changes in depression. As for the dose of LPS, accurate conclusions cannot be drawn due to the limited number of literatures, but it is clear that 0.5mg/kg, 0.83mg/kg and 1mg/kg LPS can stably induce depression behavior in mice.

## 4 Discussion

### 4.1 Mouse strains

Rodent research has long been utilized to model affective and immune deficits, as well as their interplay. Mice as experimental subjects have the sensitivity, validity, and reliability suitable for depression modeling, which can greatly provide a feasible experimental reference and develop relevant standards. Mice appear to be more suitable than rats for application in models of neuroinflammation-induced depression. The selection of appropriate mouse strains is important for determining the antidepressant activity of drugs. After the frequency analysis of the mouse strains selected in the 168 included studies, we found that C57BL/6 mice (inbred strain) were used in many studies, followed by ICR mice (closed colony), Swiss mice (outbred strain), and Balb/c mice (inbred strain). Baseline immobility time can more appropriately describe innate vulnerability to stressors and the tendency to despair under stress (31). Different genetic backgrounds can modify responses through different baseline levels of behavior. Lucki compared outbred and inbred mice by FST and suggested that mouse strains with higher immobility values, such as C57BL/6, can be more susceptible to stress-induced depressive behavior (30). Importantly, many studies have shown that mouse strains differ in their sensitivity to antidepressants and that the behavioral effects and mechanisms of action of drugs differ between strains (30, 32, 33). Study have shown that outbred mice are more variable in their baseline performance than inbred strains (30, 34), which can contribute to some of the high heterogeneity in our experimental results. Moreover, strains with less variation in baseline C57BL/6 can provide more accurate changes in drug efficacy (30). Different strains of mice showed similarities in baseline immobility in TST and FST, and Nadège Ripoll’s study showed that C57BL/6 mice had a longer baseline immobility time in the hanging tail test compared with other strains (Swiss, NMRI, DBA/2) (35). However, some studies have shown the opposite results. David D J P did not observe baseline differences in FST among Swiss, NMRI, DBA/2, and C57BL/6J Rj strains (36).

Based on the results of the meta-analysis, C57BL/6 mice produced lower effect values under LPS induction than ICR and Swiss mice. However, in general, C57BL/6 mice had lower heterogeneity and could consistently model depression and respond to drug effects, which is consistent with the conclusions drawn earlier. Alternatively, it is well known that there are important differences in the behavior of mice of the same strain purchased from different providers. Therefore, we recommend that the selection of a suitable animal strain for establishing a depression-like model should be taken into account based on various factors such as the purpose and content of the study, drug properties, and experimental conditions, and ensure that the mice were derived from the same providers or laboratory production.

### 4.2 LPS administration

The induction of cytokines by the low-dose systemic administration of LPS can induce depressive features. Initially, LPS acts as a pathogen-associated molecular patterns (PAMP) that induces peripheral inflammation to produce pro-inflammatory cytokines. PAMP and circulating cytokines act on toll-like receptors in macrophage-like cells to transmit peripheral inflammatory signals to the center by synthesizing and releasing central cytokines (37, 38). The effects of peripheral pro-inflammatory cytokines can also be transmitted to the brain, leading to microglia activation and pro-inflammatory cytokine release (39). At about 6 h, the production of pro-inflammatory cytokines leads to disease behaviors, including fever, anorexia, decreased exercise capacity, and reduced social interaction; however, it is usually terminated by endogenous anti-inflammatory molecules (40). When the production of pro-inflammatory cytokines continues, and the amount of anti-inflammatory cytokines produced is insufficient to counteract the amount of pro-inflammatory cytokines, depression-like behaviors are induced and peaked at about 24 h of LPS injection (41). Genetic background and social environment affect depression-like behaviors induced by a single IP of LPS (42).

#### 4.2.1 Routes of LPS administration

Studies in the literature included in the present study have shown three common types of LPS administration: IP, ICV, and IG, with IP being the most commonly used mode of administration. Intraperitoneal injection of LPS induces peritoneal inflammation and can modulate the activation of the brainstem, hypothalamus, and limbic structures *via* vagal afferents in response to peripherally administered LPS, thus inducing depression-like behaviors at about 24 hours. Moreover, IP injection is simple and reproducible and is therefore selected as the most commonly used method for LPS-induced depression. The direct injection of LPS into the brain can cause intracerebral inflammation, which is closely associated with psychiatric disorders, cognitive dysfunction, and motor impairment. The ICV injection of LPS caused mice to exhibit depressive states in the Y-maze to detect spatial memory and TST to detect despair, induced dendritic atrophy in the prefrontal cortex and hippocampal pyramidal neurons (43), and increased the levels of the pro-inflammatory cytokines IL-1β and TNF-α in the hippocampus, leading to neuronal damage. The intracerebral injection of LPS could stably establish a depression model. However, there are shortcomings in this complicated operation which cause extensive damage to experimental animals and have high mortality.

#### 4.2.2 Times and doses of LPS administration

Single or multiple LPS injections represent acute or chronic LPS-induced depression, respectively. Various studies have shown that both acute and chronic LPS excitation can successfully induce depressive behaviors in rodents (44–47). Acute LPS administered systemically in mice or rats release pro-inflammatory cytokines that induce strong but transient disease behaviors as evidenced by reduced motor activity and weight loss (48, 49). FST, SPT, and OFT are generally performed to assess depression-like models 24 hours after LPS injection. study has shown that depression-like behaviors can still be observed in mice 48 h after LPS injection (50). Moreover, depression is a chronic and recurrent disease characterized by persistent levels of inflammatory markers (48); hence, Depression models were derived by multiple repeated LPS injections to mimic persistent inflammation. Multiple LPS injections were further divided into single-dose multiple injections and multiple-dose injections. Yong He (49) compared acute and chronic LPS depression mouse models with a protocol of single and multiple injections (once daily for 7 days) of 0.5 mg/kg LPS and showed that both administration methods were successful in establishing depression models; however, the acute LPS-induced mice showed significantly more depression-like behaviors in SPT and FST than the chronic LPS-induced mice (49). Robin A. Wickens (48) compared repeatedly increasing doses of LPS (0.1, 0.42, and 0.83 mg/kg) with a constant dose (0.83 mg/kg) for 3 days, and with increasing doses, mice developed disease behaviors; however, they also developed tolerance to repeated constant doses of LPS, resulting in diminished behavioral responses (48).

The serotype has an important influence on the dose selection of LPS. We analyzed the frequency of the use of LPS serotypes and found that 055: B5 was the most frequently used model, followed by 0127: B8 and 0111: B4. We found no significant difference in the frequency of selecting these three serotypes. However, a selective difference in the dose of LPS selected for different serotypes and a significant effect on depressive behaviors or biochemical indicators must be systematically evaluated. Different LPS serotypes induce the differential activation of inflammatory transcription factors, such as IL-1β, IL-6, interleukin-8 (IL-8), and TNF-α, leading to differential protein expression, which may be related to the structure of LPS models, and the selective use of LPS serotypes may help investigate activation-specific inflammatory mechanisms (51, 52).

The meta-analysis results showed that the three doses of the single injection of LPS at 0.5 mg/kg, 0.83 mg/kg, and 1 mg/kg induced depression models in mice and were closely related to the despairing behaviors of animals. Mice in the model group were immobilized for a longer period than those in the FST compared with those in the control group. High heterogeneity was observed in some single-group analyses, probably indicating difficulties in replicating the LPS depression model using these programs. The effects of other factors, such as mouse strain, individual differences (weight, age, and sex), environment (water and food, light, temperature, and noise), experimental design (sequence of FST and other behavioral tests, water temperature of FST test, and test time), on the experimental results were considered.

### 4.3 Behavior test

Among the 168 papers included in the present study, there were 18 behavioral tests, and the most commonly used methods to evaluate depression-like behaviors in mice were FST, TST, OFT, and SPT, in that order. FST and TST have a similar theoretical basis and measure of time spent stationary in an inescapable environment; the immobility state reflects despair-like inhibitory learning behaviors and is an important indicator of depressive psychomotor retardation (53). The effects of LPS usually disappear at higher sucrose concentrations, which are commonly used in 1% sucrose solutions (54); OFT simulates an unsafe environment to assess the autonomous behavior of an animal to reveal the level of tension in the animal. When animals are afraid of a new environment, they tend to move around the edge area of the open field box and rarely in the central area. Additionally, locomotor activity and autonomic activity tests were performed to assess the motor ability of mice (55). Y maze, Morris water maze (MWM) (56), and elevated plus maze (EPM) (57) tests were performed to assess spatial learning memory and the ability to explore new environments, whereas the novel object recognition test (NORT) assessed non-spatial memory in mice (58). The novelty suppressed-feeding test (NSFT) is an observation of the conflicting behavior of animals in a hunger state with the desire to ingest food and the fear of entering a bright central area, and it assesses depressive or anxious behaviors as an indicator of food intake and latency to find food. The social interaction test (SIT) is performed to evaluate social competence in mice, and the splash test (ST) is a valid marker for assessing self-care behaviors in depressed mice.

FST is the most commonly performed behavioral experiment for evaluating depression models and screening antidepressants in preclinical studies because of its ease of operation and sensitivity. Many published papers have described the experimental protocols and considerations of FST (59–61). However, the FST results were susceptible to many external (experimental design and environmental conditions) and biological factors (strain, weight, age, and individual differences). The FST experimental protocol, including the size of the test bucket, water depth, water temperature, and test time, affected the experimental results. The size of the test barrel affected FST immobility. Sunal et al. placed mice into vertical Plexiglas cylinders (height: 30 cm, diameter: 10, 20, 30, and 50 cm) in 20-cm deep water to swim, and as the diameter of the barrel increased, FST immobility in mice decreased (62). Compared with other barrels, the total duration of immobility was shorter, and the latency period was longer in the 10-cm test barrel (63), the most commonly used test barrel size for mice. The water temperature during the test was also an important affecting factor, with mice exhibiting greater immobility when swimming at 25°C than at 35°C (64), and reduced swimming speed was observed in the MWM test at low temperatures (65). However, the effect of water temperature on the FST results was different in different mouse strains, as the immobility of C57BL/6 mice increased when the water temperature increased; however, BALB/C mice showed the opposite performance (66). Our extensive literature analysis showed that most investigators selected a water temperature of 21–25°C for the FST. In addition, the mice showed a clear 24-h rhythm of immobility in the FST, with a shorter duration of immobility in mice at noon (12:00 PM–2 PM) than at midnight (12:00 AM–2:00 AM). If more than one behavioral test is required, we recommend the FST as the last test. In addition, studies have shown that environmental factors such as light, light/dark cycle time, noise, odor, and living environment can affect the immobility time of animals (63).

It has to be mentioned that the interpretation of duration of immobility as measure of behavioral despair has been criticized repeatedly (67). Hawkins et al. (68) agree with the use of the FST as an innovative antidepressant screening tool, but refute the notion that immobility in the FST represents hopelessness. The researchers (69) believe that immobility is an adaptive response to an inescapable environment, rather than depression-like behavior. Nonetheless, its high efficacy has made it a popular behavioral test for selecting antidepressants.

## 5 Conclusion

Depression is a complex biological heterogeneous disorder. Due to differences between animal models and human disease groups, it is challenging to replicate the human depression phenotype in a depression-like animal model. LPS-induced models of depression in mice are subject to genetic, environmental, and experimental parameters that may ultimately have a qualitative impact on the study results obtained by applying this model. Therefore, reaching consensus on modeling methods or experimental parameters in depressive-like animal models is essential to avoid unnecessary external effects. Our results suggests that mice strains and LPS administration play a key role in the assessment of behavioral outcomes in these models. This provides an important reference for the rational application of such models. In addition, the design, implementation, measurement criteria, and reporting of this animal model need to be further improved and standardized to facilitate the development of better animal experiments and clinical studies of inflammation induced depression. Compared with the traditional animal model of depression, LPS model has some disadvantages in that it cannot more fully replicate the depression phenotype. Rodents were used to identify animal models of antidepressants commonly associated with stress or genetic factors. However, we believe that the use of depression-like animal models based on neuroinflammation is beneficial for further elucidating the mechanism of action of antidepressants. Finally, although there has been controversy surrounding the use of animal models, their utility and value in depression research over the past decades cannot be ignored. Optimizing experimental protocols is one of the means to reduce the harm to animals.

## Author contributions

RY, KZ, and YX contributed to the conception, design, and preparation of the manuscript. YL, ZT, and RZ outlined the initial literature review and extracted literature data. YM, NL contributed significantly to drafting the manuscript and critically revising it, GL, ZC, PG, NL meticulously guided the application of the methodology. All authors contributed to the article and approved the submitted version.
